# Application of a Double- and Triple-Stacked Hexapod (Taylor Spatial Frame) for High-Energy Segmental Tibial Fractures

**DOI:** 10.7759/cureus.88834

**Published:** 2025-07-27

**Authors:** Thylane E Vancastell, Sander V Ianniello, Joseph Muscat, Kareem Edres, Matija Krkovic

**Affiliations:** 1 Trauma and Orthopaedic Surgery, Addenbrooke's Hospital, Cambridge University Hospitals NHS Foundation Trust, Cambridge, GBR; 2 Trauma and Orthopaedics, The Queen Elizabeth Hospital King's Lynn NHS Foundation Trust, King's Lynn, GBR; 3 Trauma and Orthopaedics, Addenbrooke's Hospital, Cambridge University Hospitals NHS Foundation Trust, Cambridge, GBR

**Keywords:** double-stacked tsf, external fixator, fine wire frame, hexapod, high-energy segmental tibial fractures, lower leg segmental fracture, taylor spatial frame, tibial fracture, triple-stacked tsf, tsf

## Abstract

Segmental open tibia fractures from high-energy trauma present significant challenges in achieving stability, alignment, as well as soft tissue preservation. Conventional internal fixation methods often pose risks of infection, non-union, and soft tissue compromise. The Taylor Spatial Frame (TSF), a hexapod-based external fixator, offers multi-planar correction and gradual deformity control, which can prove instrumental in complex segmental fractures.

This technical note details the application of double- or triple-stacked TSFs to treat multi-level tibial fractures, ensuring independent correction of each segment while maintaining limb stability.

The technique includes wire and pin placement strategies, frame synchronization, and computational correction. It provides a versatile and adaptable solution for managing challenging segmental tibia fractures.

## Introduction

Segmental tibial fractures often result from high-energy trauma and require complex reconstruction strategies due to their instability, high risk of non-union, and extensive soft tissue injury [1-4]. Traditional intramedullary nailing or plating techniques may not be viable due to bone loss, infection risk, or poor soft tissue conditions [2-4].

The Taylor Spatial Frame (TSF), a hexapod external fixator, provides a six-degree-of-freedom correction, allowing for precise control over multi-planar deformities and segmental transport [4,5]. Six-degree-of-freedom correction refers to the ability to precisely adjust bone position in three translational (X, Y, Z) and three rotational (pitch, roll, yaw) planes to achieve accurate deformity correction. In cases of segmental fractures, the use of a single TSF may not provide adequate control over each fracture zone [4]. Instead, utilizing multiple hexapods in a synchronized configuration allows for independent control of each segment, facilitating optimal alignment and staged deformity correction [4].

This technical report describes the surgical technique, wire and pin configuration, frame setup, and postoperative protocol for treating a high-energy segmental tibial fracture using double- (for two fracture lines) or triple- (for three fracture lines) stacked hexapod external fixators.

## Technical report

Preoperative planning

A thorough preoperative evaluation is essential to determine the fracture pattern, soft tissue status, and feasibility of bone transport, particularly in cases involving segmental bone loss. For clarity, this technical note aims to describe only cases of multisegmental tibial fractures without bone loss. This planning phase ensures precise alignment, stabilization, and correction strategies are in place before surgical intervention.

The radiographic evaluation includes standard anteroposterior (AP) and lateral X-rays to assess the fracture configuration and alignment of the tibia.

**Figure 1:**
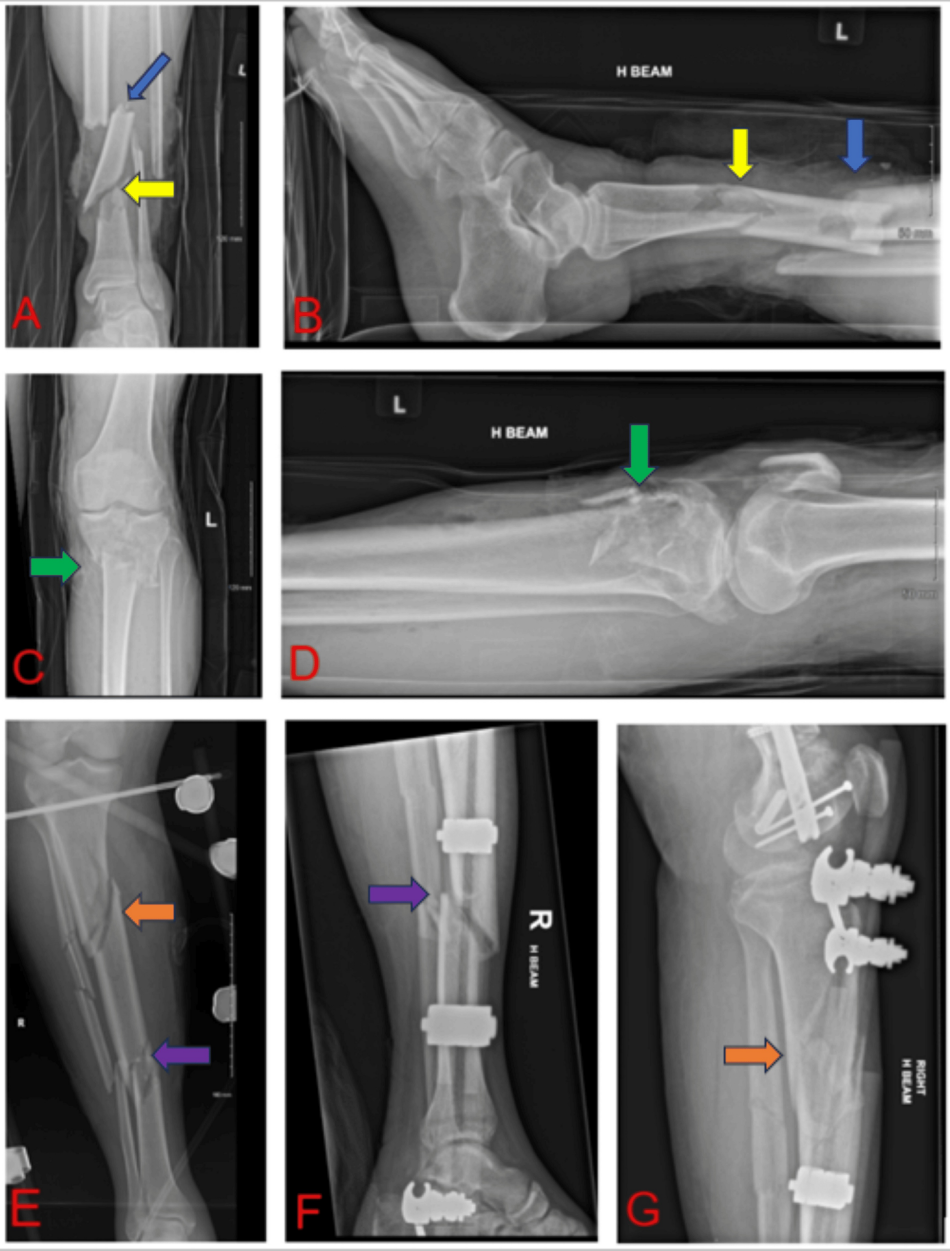
Radiographs of multisegmental tibial and fibular fractures in two separate patients, obtained following injury and prior to the surgical application of a multi-stacked Taylor Spatial Frame. In the first case (A–D), three distinct fracture lines are visible in the left tibia, necessitating correction at three levels. This configuration requires the use of a triple-stacked hexapod fixator. In the second case (E–G), radiographic imaging was performed following initial surgical stabilisation using an external fixator. The right tibia demonstrates multisegmental fractures requiring correction at two main levels, for which a double-stacked hexapod is indicated. A: Anteroposterior radiograph of the left distal tibia and fibula demonstrating the distal and middle fracture lines, comprising two of the three main fracture sites (yellow arrow: distal fracture line; blue arrow: middle fracture line) B: Lateral radiograph of the left distal tibia and fibula showing the distal and middle fracture lines, part of a tri-segmental fracture configuration (yellow arrow: distal fracture line; blue arrow: middle fracture line) C: Anteroposterior radiograph of the left proximal tibia and fibula illustrating the proximal fracture line, representing the third of three main fracture sites (green arrow indicates the proximal fracture line) D: Lateral radiograph of the left proximal tibia and fibula depicting the proximal fracture line of the three-segment tibial fracture (green arrow indicates the proximal fracture line) E: Anteroposterior radiograph of the entire right tibia and fibula providing an overview of the two main fracture sites (purple arrow: distal fracture line; orange arrow: proximal fracture line) F: Lateral radiograph of the right distal tibia and fibula demonstrating the distal fracture line, one of two primary fracture sites (purple arrow points to the distal fracture line) G: Lateral radiograph of the right proximal tibia and fibula showing the proximal fracture line of the bi-segmental fracture pattern (orange arrow shows the proximal fracture line) ^_These radiographic images were obtained in our hospital during the course of the patients’ treatment. Informed consent was obtained for their publication. The patients are under our care in the limb reconstruction unit, and the surgical procedures were performed by two of the authors as part of the overall treatment plans._^

Figure 1 shows three main fracture lines as an example of a triple-stacked TSF (Figures 1A-1D) as well as two main fracture lines as an example of a double-stacked TSF (Figures 1E-1G). In more complex cases, a computed tomography (CT) scan is performed to provide detailed fracture mapping and three-dimensional reconstruction, enabling a more comprehensive understanding of the bone defect and deformity. Depending on the fracture pattern, we typically do not perform a CT scan in clearly defined two- or three-part fractures. However, a CT scan is indicated when there is multifragmentary comminution within individual segments, and when fracture lines are unclear and their extent is difficult to assess.

Since soft tissue integrity plays a crucial role in managing open fractures and fractures with compromised soft tissue, a thorough assessment of soft tissue is conducted, particularly for injuries classified as Gustilo IIIb or IIIc [6]. In cases where extensive soft tissue damage is present, consultation with plastic surgery specialists is necessary to determine whether flap coverage will be required as part of the treatment plan.

We do not undertake preoperative planning of the deformity correction using the TSF software [7]. Instead, our aim is to position the TSF rings intraoperatively so that they are perpendicular to the bone fragments in both the anteroposterior and lateral views. We plan gradual deformity correction postoperatively, based on the initial postoperative X-ray, and commence correction once the frame prescription has been completed. This approach reduces theatre time and radiation dosage for the patient and the surgeon.

Surgical steps

Step 1: Initial Debridement and Temporary Stabilization

In cases of open fractures, our department follows the British Orthopaedic Association & British Association of Plastic, Reconstructive and Aesthetics Surgeons (BOAST) guidelines for open fracture management, which includes a joint orthoplastic approach to initial debridement and stabilisation. The timing of the debridement is guided by the energy of the injury and the degree of contamination [8]. In cases where there has been significant contamination, periosteal and soft tissue damage (Gustilo-Anderson III a and above [6]), the bone ends are aggressively debrided with an oscillating saw or osteotome to remove all devitalised and contaminated bone. The interim stabilisation of the bone is commonly achieved through external fixation.

Following the debridement, if definitive soft tissue coverage is not possible, a vacuum-assisted closure (VAC) dressing is applied as a form of negative pressure wound therapy (NPWT) to cover the soft tissue defect [9]. We aim to achieve definitive soft tissue coverage, in the form of a local or free flap, within 72 hours post-injury. Patients are continued on intravenous antibiotic prophylaxis following local guidelines until this is achieved [8].

Step 2: Application of Double- or Triple-Stacked Hexapod External Fixators

This outlines the application of a double or triple-stacked hexapod for multisegmental tibia fractures. Fractures with significant bone loss that require bone transport are outside the scope of this technical note.

The timing of the application of the TSF is guided by the healing time for the soft tissue coverage and the absence of infection. We allow a period of two to three weeks for local flaps and up to four weeks for free flaps to ensure adequate flap integration before applying the TSF.

**Figure 2:**
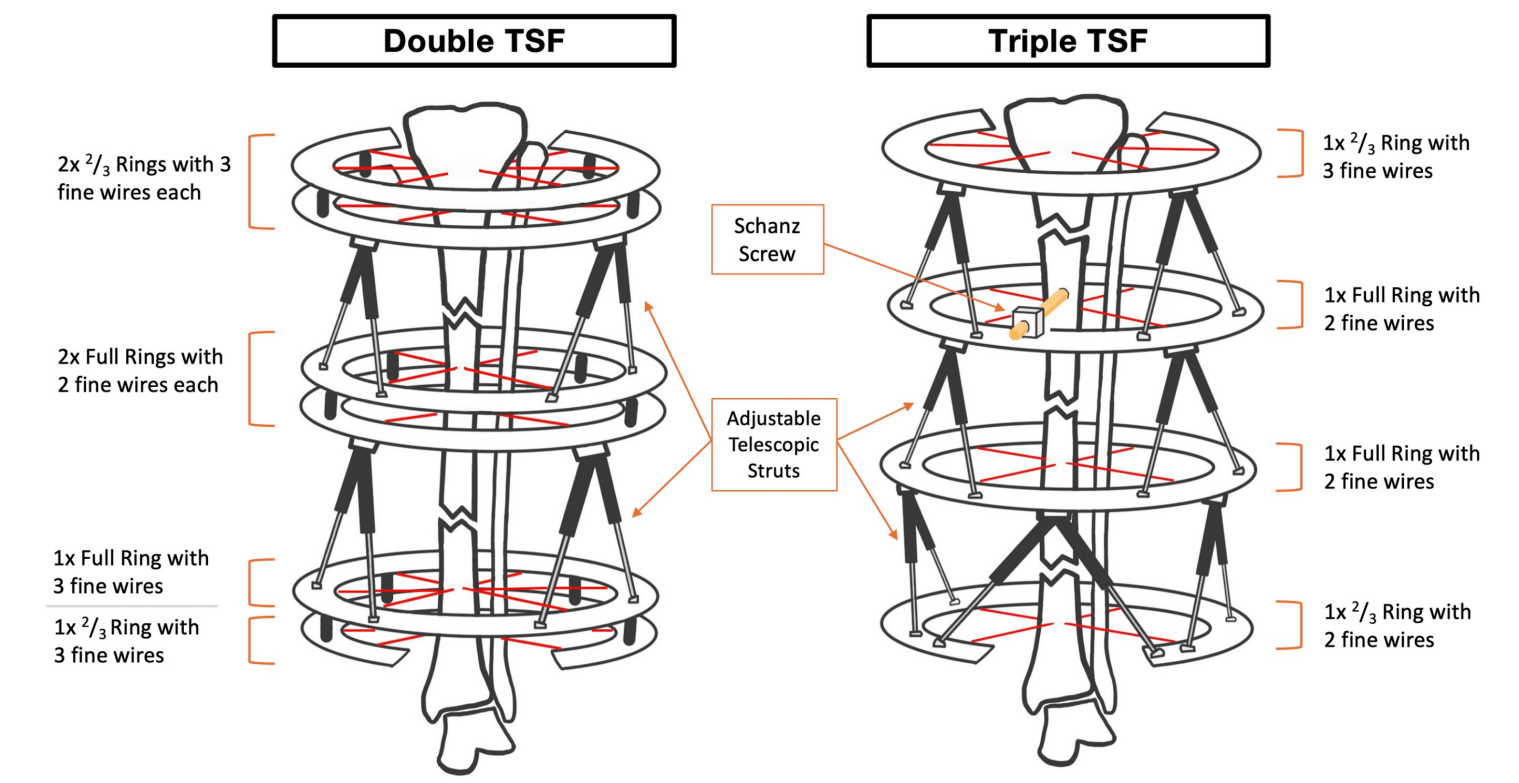
Schematic of double- and triple-stacked Taylor Spatial Frame (TSF) constructs for the management of two and three fracture lines, respectively The rings are secured to the tibia using 1.8 mm bayonet-tip olive wires. The number of wires per ring may vary as per the diagram, depending on structural needs. The adjustable telescopic struts span across the fracture line to allow manipulation of the individual fractured segments along multiple axes. These movements are achieved through daily micro-adjustments guided by the TSF software [7]. The triple TSF construct may include a Schanz screw (also known as a half-pin) for additional stability. Note that only a few adjustable telescopic struts are depicted for visual clarity of the schematic, and that in practice, six or more struts per frame may be applied. ^Schematics were created by the authors themselves using “Goodnotes 6” software (version 6.6.49).^

It is important to determine the number of bone segments before the TSF application. A segment, in this technical example, is defined as a section of bone above, below, or between a fracture line. Therefore, a segmental tibia fracture with two main fracture lines has three segments, each of which will need to be addressed with ring application to form a double-stacked TSF. Similarly, a segmental fracture with three main fracture lines will have four segments, each of which will require a ring application to form a triple-stacked TSF. The schematic image below illustrates double- and triple-stacked TSFs, corresponding to the number of fracture lines requiring correction (Figure 2).

**Figure 3:**
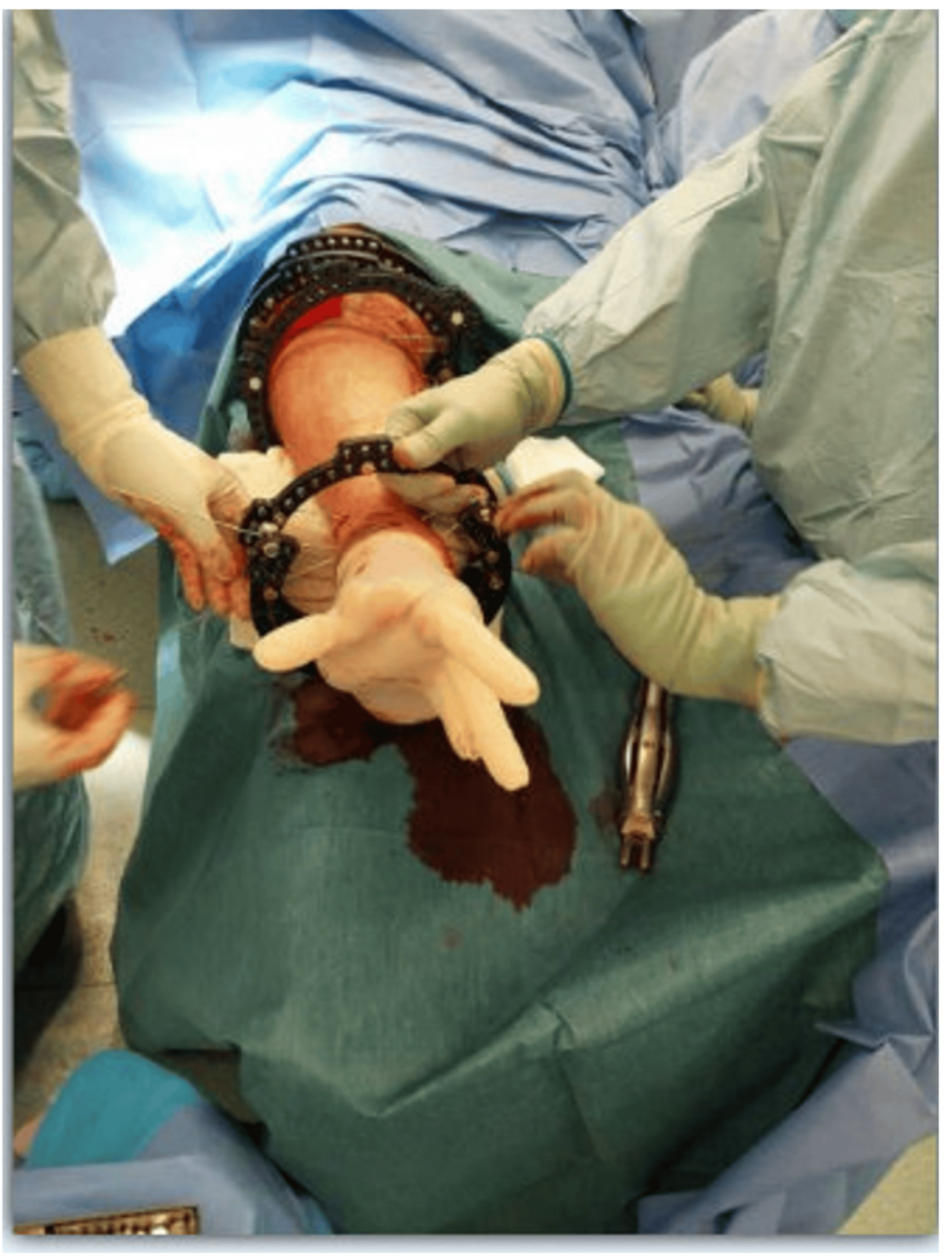
Intraoperative photograph demonstrating circular fine-wire frame ring fitting Each full ring is assembled from two appropriately sized half-rings, joined using bolts and nuts to accommodate the patient’s leg. For the two most proximal and one or two most distal levels, 2/3 rings are utilised, with the openings oriented posteriorly proximally and anteriorly distally—to allow unhindered knee and ankle movement, respectively. ^This intraoperative photographic image was obtained by the authors with the patient’s informed consent for both image acquisition and publication. The patient is undergoing treatment in our limb reconstruction unit, and the surgical procedure was performed by two of the authors as part of the management under our care.^

The double- or triple-stacked hexapod external fixator constructs are assembled directly on the leg during surgery and applied sequentially to allow independent control of each fracture segment while maintaining overall stability and alignment (Figure 3).

Determining the level of the rings is achieved using fluoroscopic guidance while the patient is supine on the operating table. The approximate level of the rings and struts is marked using a skin marker. Care is taken to ensure the rings are parallel to the ankle joint.

Application of the Rings to the Proximal Segment

Two rings are positioned on the proximal segment. This is often in the form of two 2/3 rings with the opening oriented posteriorly to allow for knee flexion. Each ring is fixed with three 1.8 mm olive-tipped guidewires. Two olive-tipped guidewires were inserted in a crossed configuration, with an inter-wire angle ranging from 30 to 90 degrees. The addition of a third wire prevents translational movement at the point of convergence of the crossed wires, while also reducing the risk of neurovascular injury associated with perpendicularly placed wires in the anteroposterior and lateral planes and strictly sticking to the safe corridors [10].

A second ring is applied below in a similar manner and connected to the first ring using threaded sockets.

Application to the Middle and Distal Segments

Each segment requires at least one ring to provide control. The decision to apply one or two rings to each bone segment is primarily determined by the length of the segment and the distance to the adjacent segment's ring. The double-stacked hexapod for a segmental (two main fracture lines) fracture requires at least one further full ring to the middle segment and a 2/3 ring to the distal segment, with the opening orientated anteriorly to allow for ankle dorsiflexion.

The triple-stacked hexapod for a multisegmental (three main fracture lines) fracture will require the application of a full ring to both middle segments and a 2/3 ring to the distal segment. These rings are secured with two olive-tipped wires each in a crossed fashion as described above, without the use of a third wire.

In a triple-stacked TSF construct, the distal ring of the proximal hexapod segment serves as the proximal ring of the middle hexapod segment, which spans the diaphyseal fracture and attaches to two adjacent bone segments for precise control of the fracture fragments.

In a double-stacked hexapod construct, the distal ring of the proximal frame functions as the proximal ring of the distal frame, which is affixed to the distal tibia using a similar six-wire construct to ensure stability and anatomical alignment. To maintain optimal function, each frame is aligned independently while ensuring that no mechanical interference occurs between them. Cross-pin fixation is employed at each segment to optimise angular and rotational stability. If pins from the spanning external fixator are stable and suitably positioned for integration into the TSFs, they are retained and incorporated into the frame.

Although tensioned wires serve as the primary stabilising elements, half-pins are utilised when greater corrective force is anticipated, particularly in cases of predominant apex anterior or posterior deformities, or when a single ring with only two wires is applied to a bone fragment.

Minimal correction is performed intraoperatively to minimize operating room and anesthesia duration, thereby reducing associated risks such as infections, patient hypothermia, and radiation exposure for both patients and surgical staff, while allowing for more accurate and precise postoperative deformity correction using X-rays in conjunction with TSF software postoperatively. It is common for patients to leave the operating theatre with apparent limb malalignment, which is not considered problematic. Gradual correction is performed postoperatively using frame adjustments based on prescribed protocols [7].

Securing and Tensioning of the Wires

**Figure 4:**
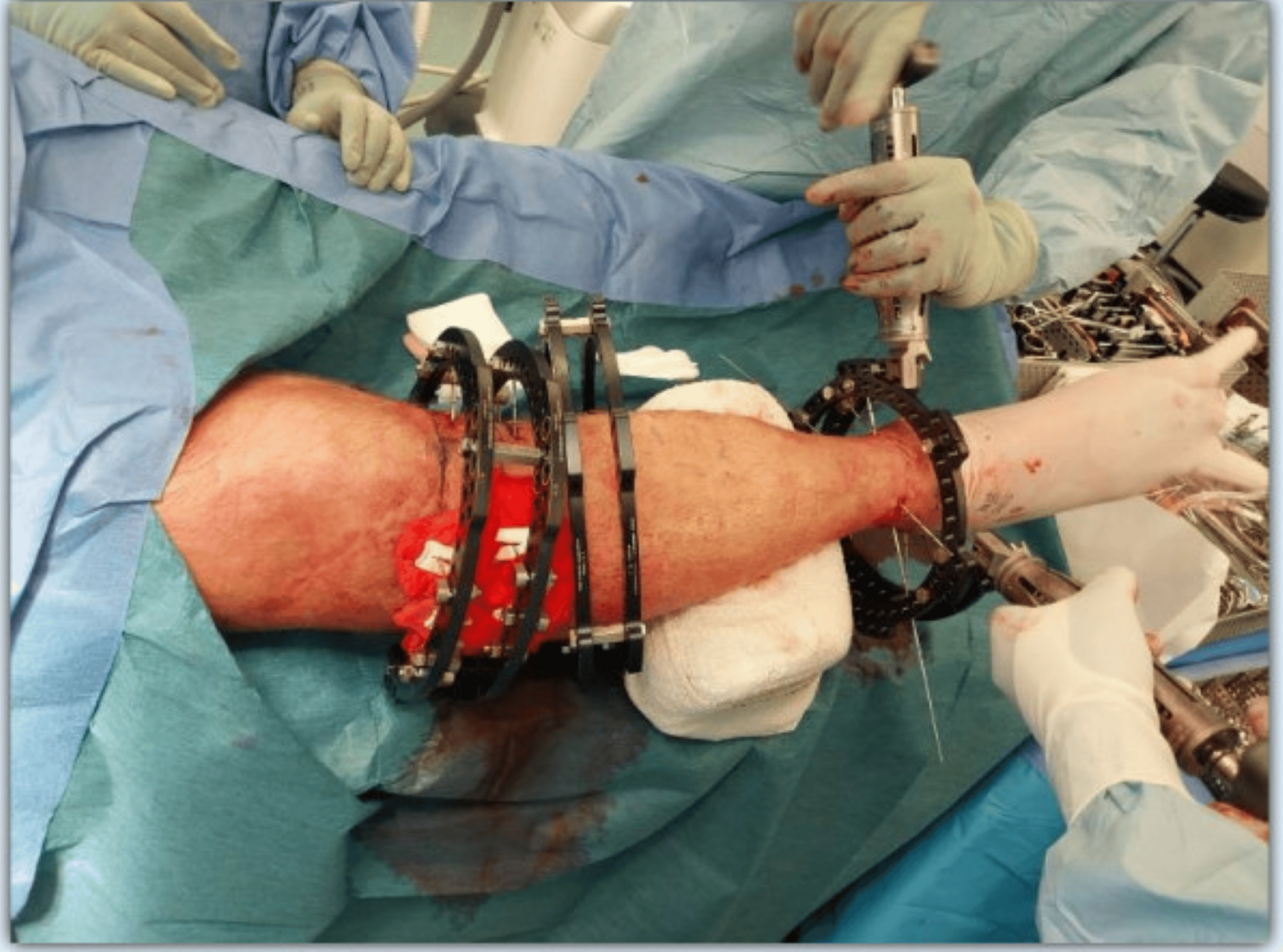
Intraoperative imaging depicting the wire tensioning process during the application of the Taylor Spatial Frame. After insertion of the 1.8 mm bayonet-tip olive wires under fluoroscopic guidance, the wires are tensioned using a specialised wire tensioner. This device allows precise application of tension, typically up to 110 Nm, ensuring optimal wire tightness for mechanical stability. The tensioners are attached to the wires bilaterally and gradually tightened. Once the desired tension is achieved, the wires, which are already secured to the ring using slotted bolts and nuts, are finally tightened with spanners, maintaining the required stability throughout the procedure. ^This intraoperative photographic image was obtained by the authors with the patient’s informed consent for both image acquisition and publication. The patient is undergoing treatment in our limb reconstruction unit, and the surgical procedure was performed by two of the authors as part of the management under our care.^

The 1.8 mm bayonet-tip olive wires are inserted under image intensifier control, secured to the rings using slotted bolts and nuts, which are tightened to 110 Nm (Newton metres) with the wire tensioner. The bolts and nuts are finally tightened with spanners (Figure 4). Following tensioning, the free ends of the wires are broken off to mitigate injury from the sharp wire ends.

Application of the Struts

**Figure 5:**
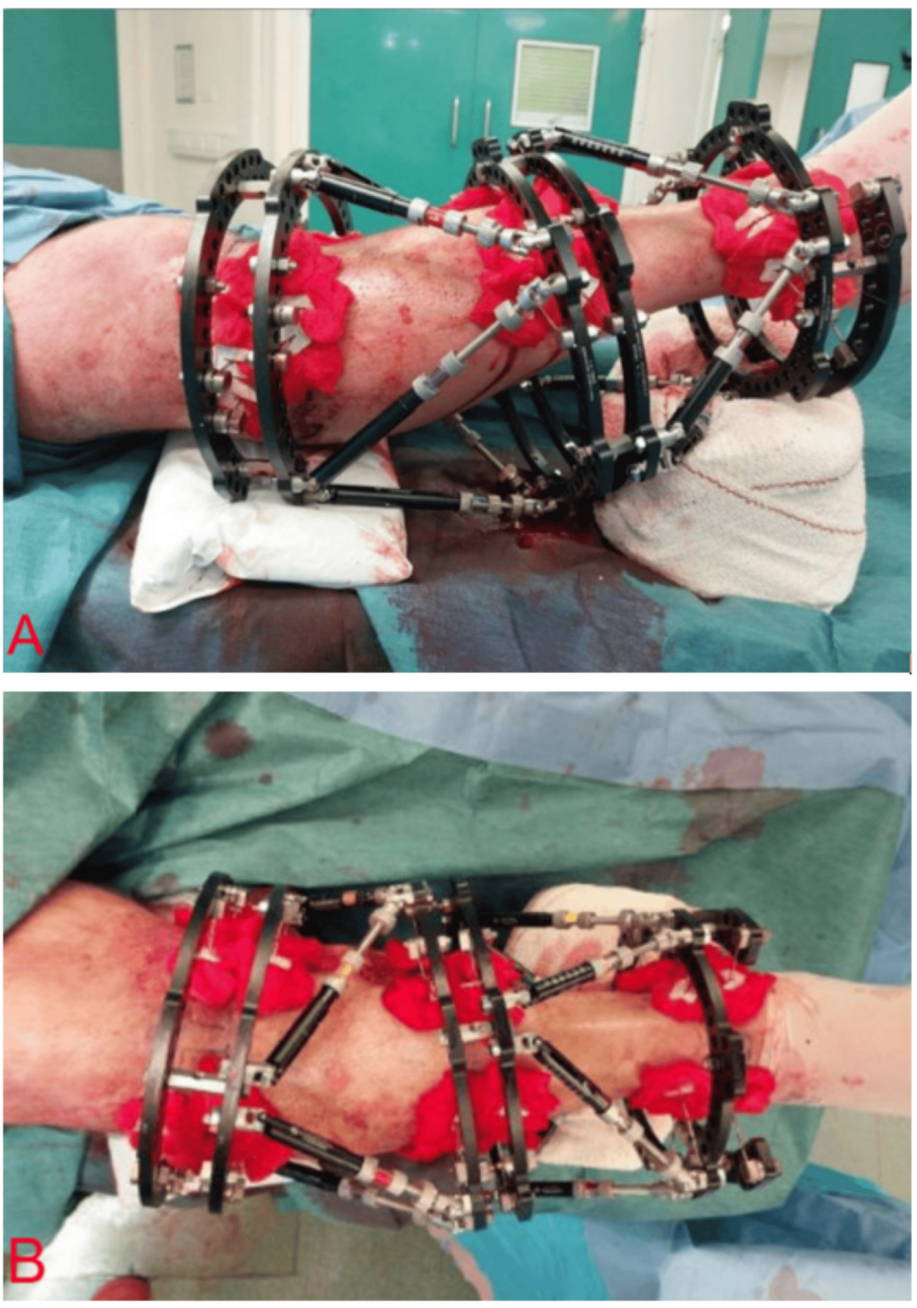
Intraoperative photographs following complete application of a double-stacked Taylor Spatial Frame (TSF), securely affixed to the tibia A: Lateral view demonstrating the final alignment of the rings and struts relative to the bone segments, illustrating precise construct positioning in the sagittal plane at the conclusion of the procedure. B: Anterior view showing the fully assembled double-stacked TSF mounted on the tibia. This perspective is essential for confirming correct ring placement and overall frame symmetry. It should be noted that no major alignment corrections are performed intraoperatively; initial frame application is intended solely to stabilise the fracture segments. Controlled, gradual deformity correction is subsequently performed postoperatively using TSF software to ensure accurate and safe adjustment. ^These intraoperative photographic images were obtained by the authors with the patient’s informed consent for both image acquisition and publication. The patient is undergoing treatment in our limb reconstruction unit, and the surgical procedure was performed by two of the authors as part of the management under our care.^

Six appropriately sized hexapod struts are applied to each segment in the conventional manner. The primary objective of the initial positioning is to achieve stable fixation of the fracture segments (Figure 5). Controlled and gradual deformity correction is then performed postoperatively using the TSF software to ensure accurate and safe adjustment [7].

**Figure 6:**
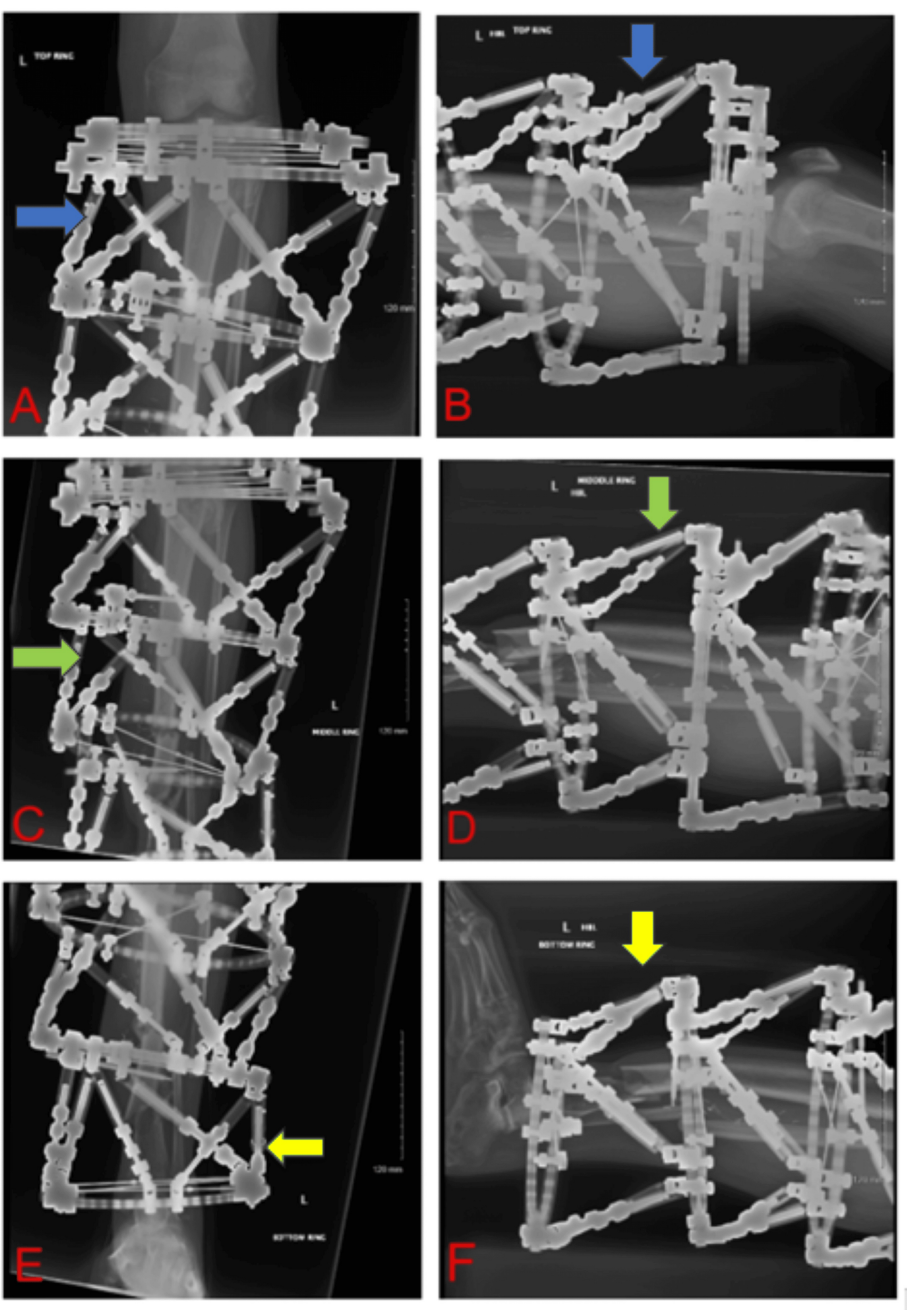
Postoperative radiographs showing a triple-stacked Taylor Spatial Frame (TSF) applied, with alignment correction planned using TSF software Following the initial postoperative radiographs, alignment correction is initiated using the TSF software. For each segment mounted with a hexapod that contains a primary fracture line, radiographs must be obtained in two orthogonal planes: anteroposterior and lateral. In each instance, the central X-ray beam must be oriented perpendicular to the fracture plane. Consequently, a total of four radiographs is required for double-stacked constructs, and six for triple-stacked configurations [7]. All images presented are radiographs of the affected left tibia treated with a triple-stacked hexapod external fixator. A: Anteroposterior radiograph focused on the proximal hexapod segment of the left tibia (blue arrow). B: Lateral radiograph of the proximal hexapod segment of the left tibia (blue arrow). C: Anteroposterior radiograph centred on the middle hexapod segment, depicting the tibial shaft fracture (green arrow). D: Lateral radiograph focusing on the middle TSF rings and struts and their fixation to the tibial shaft segments (green arrow). E: Anteroposterior radiograph of the distal hexapod segment at the distal tibia and ankle level (yellow arrow). F: Lateral radiograph of the distal hexapod segment at the distal tibia (yellow arrow). ^These radiographic images were obtained in our hospital during the course of the patient’s treatment. Informed consent was obtained for their publication. The patient is under our care in the limb reconstruction unit, and the surgical procedures were performed by two of the authors as part of the overall treatment plans.^

Following the first postoperative radiographs, computational adjustments are initiated using the TSF software [7]. It is essential that for each hexapod-mounted segment, each containing a primary fracture line, individual radiographs are obtained in two planes: anteroposterior and lateral. In each case, the central X-ray beam must be directed perpendicular to the fracture plane. Accordingly, four radiographs are required for double-stacked frames and six for triple-stacked frames (Figure 6). The TSF software is programmed to facilitate gradual deformity correction, providing precise control over the alignment of each bone segment [7]. Fine-tuning of the correction parameters is undertaken to optimise axial alignment, allowing for segment-specific modifications throughout the healing process. A separate frame prescription must be issued for each individual frame segment.

Post-operative management

Full weight-bearing (FWB) as tolerated is introduced immediately postoperatively. Patients are advised to shower daily.

Post-operative pain in these patients is often a concern that requires pre-operative counselling and close post-operative monitoring in both the inpatient and outpatient settings. The challenge arises in optimising analgesia requirements to allow for the patient to mobilise regularly while trying to mitigate the problems caused by long-term opiate use. Each analgesia regime may differ from patient to patient but often includes the use of a long-acting and short-acting opiate, in addition to paracetamol and ibuprofen.

We encourage immediate physiotherapy post-operatively to promote early weightbearing and prevent stiffness and contractures of the knee and ankle. Patients are reviewed at regular intervals, typically every two to four weeks, depending on the duration of the TSF prescription, the severity and complexity of the deformity requiring correction, soft tissue condition, and patient-specific factors such as mobility and pain management. On arrival, patients undergo X-ray imaging to assess correction progress, and follow-up care includes close monitoring by our specialist limb reconstruction physiotherapist to ensure optimal recovery, alignment, and function.

Frame adjustments and clinical follow-ups

**Figure 7:**
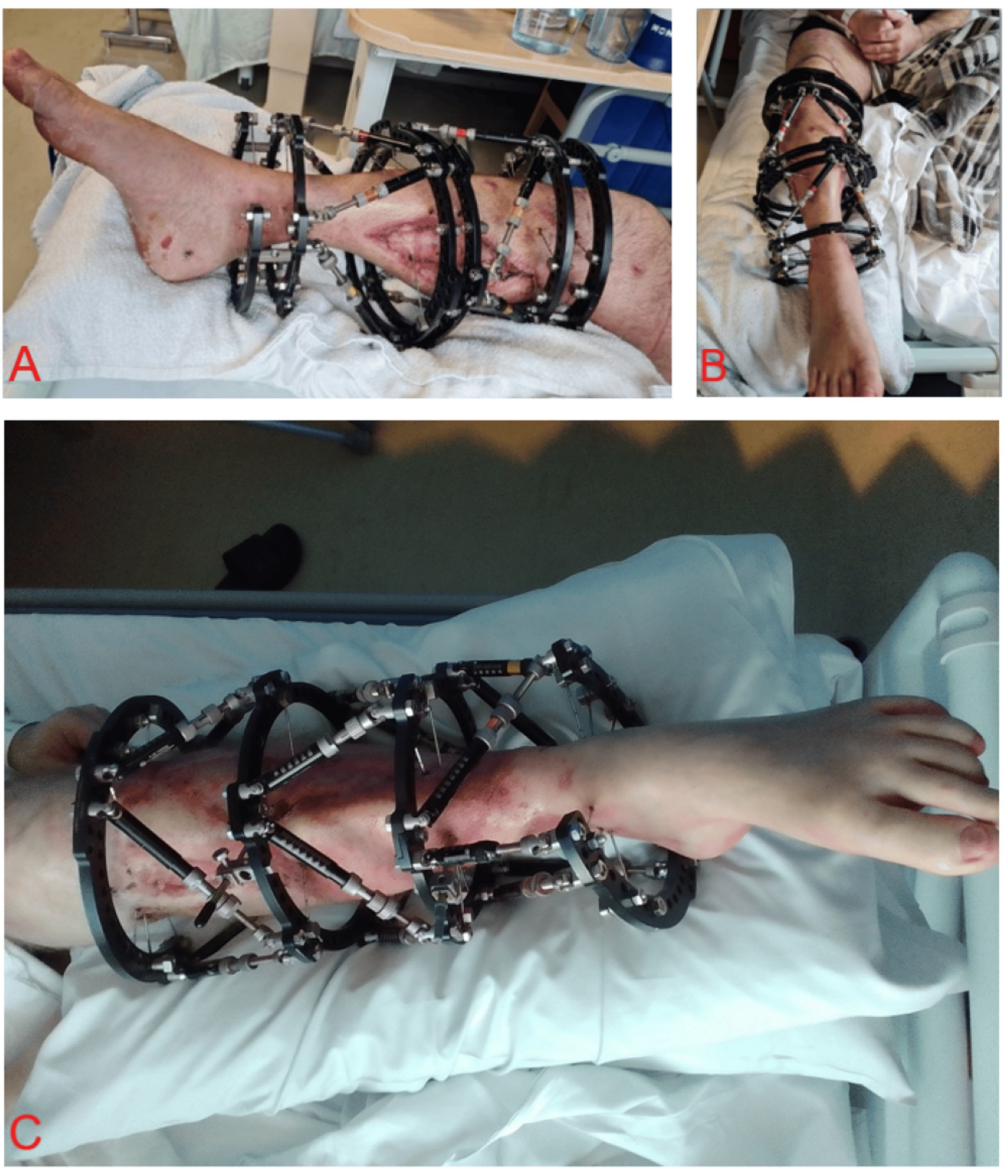
Postoperative clinical photographs demonstrating fully applied Taylor Spatial Frames (TSFs): A: A double-stacked TSF viewed medially. B: A double-stacked TSF viewed anteriorly. C: A triple-stacked TSF in situ from an anterior perspective. Struts interconnecting the structural rings are adjusted in length over time to facilitate gradual bone alignment and support bone growth and healing. ^These photographic images were obtained by the authors postoperatively on our ward with the patients’ informed consent for both image acquisition and publication. The patient is undergoing treatment in our limb reconstruction unit, and the surgical procedure was performed by two of the authors as part of the management under our care.^

Regular clinical and radiographic assessments are utilised to monitor fracture healing and alignment correction, and to issue new frame prescriptions, if required (Figure 7). Radiographs are regularly performed to allow for the early detection of alignment issues and ensure that bone correction is proceeding as planned. If adjustments are necessary, the TSF software is used to gradually modify the parameters and maintain proper alignment and mechanical stability throughout the healing process. The deformity parameters include radiograph assessment for anteroposterior, lateral, and axial angulation and translation. Rotation is determined on clinical assessment.

To prevent pin-site infections, routine pin-site care is emphasized. Patients are instructed on proper hygiene, including daily showering of the frame. We do not routinely use prophylactic antibiotics.

The duration of frame application is not fixed; frames are removed once bone union is achieved. Alternatively, they may be adjusted, partially removed, or modified as clinically indicated.

## Discussion

The use of double- or triple-stacked hexapod external fixators in the management of segmental fractures offers distinct advantages in orthopaedic trauma care. One major benefit is the independent correction of each fracture site, which significantly minimises the risk of malalignment, a common issue with conventional fixation techniques [5,11]. TSFs allow for precise, simultaneous adjustments at multiple fracture segments, ensuring optimal anatomical alignment [4].

The load-sharing biomechanics of fine-wire frames enhances the overall stability while permitting a progressive increase in weight-bearing capacity [5,12]. Unlike rigid internal fixation methods, TSFs distribute mechanical forces across the bone, facilitating natural healing while reducing implant stress [13]. The adjustable frame design enables progressive deformity correction and controlled bone transport. This feature is particularly valuable in the treatment of segmental bone defects, non-unions, and limb length discrepancies.

Additionally, the minimally invasive pin fixation reduces the risk of deep infections compared to open surgical techniques such as plating or intramedullary nailing. The percutaneous application of TSFs preserves soft tissue integrity, which is especially beneficial in high-energy trauma cases [12].

Despite its advantages, double- and triple-stacked TSFs do present a few challenges that require proactive management.

One challenge is the risk of pin-site infection, an inevitable problem with the frames' long-term use. The incidence of pin-site infection varies in the literature from 11-100%, with varying degrees of severity [14,15]. Numerous factors have been postulated to contribute to increasing rates of pin-site infection, including both patient factors and surgical factors [14]. The authors reduce the risk of pin-site infection predominantly through their surgical technique and post-operative pin-site care methods. With regards to the surgical technique, we ensure there is a stable construct with well-tensioned wire to reduce the irritation at the pin-skin interface, whilst respecting the soft tissues by ‘pushing’ the wires through until the wire is firmly on bone before drilling. Both of these factors have been shown to contribute to reducing the risk of pin-site infections [14,15].

In addition, the authors ensure that the wires do not touch the skin prior to insertion. Following the application, the wires are covered with chlorhexidine-soaked gauze kept on the pin sites for one to two days. Postoperatively, we strongly encourage daily pin-site care and showers with the frame to keep the pin sites clean. We do not routinely use antibiotics for the treatment of pin-site infections.

Another challenge is frame interference between TSFs, which can compromise the effectiveness of the fixation. This can be a particular issue with double and triple-stacked frames. To address this, careful pre-operative planning and precise frame alignment are essential to ensure adequate spacing and mechanical stability.

## Conclusions

The use of double- and triple-stacked Taylor Spatial Frame (TSF) in high-energy segmental tibial fractures allows for precise deformity correction, staged bone transport, and enhanced stability. This minimally invasive technique is an effective alternative to traditional internal fixation, particularly in cases involving bone loss, soft tissue compromise, and an increased risk of infection. The TSF has proven highly effective for segmental tibial fractures, particularly in complex or postinfectious cases. Studies consistently show high success rates, dynamic correction capability, and good functional recovery. However, challenges such as prolonged treatment duration, pin-site infections, and the need for expertise in frame adjustments must be considered.

The TSF offers a powerful solution for managing segmental fractures by providing precise correction, load-sharing biomechanics, and minimally invasive fixation. Despite challenges such as pin-site infections and prolonged treatment times, strategic management can optimize outcomes. Alternative techniques like intramedullary nailing, plating, and Ilizarov fixators each have their merits, but do not provide the same level of multi-planar correction as TSFs.
